# Human biting activity, spatial–temporal distribution and malaria vector role of *Anopheles calderoni* in the southwest of Colombia

**DOI:** 10.1186/s12936-015-0764-6

**Published:** 2015-06-24

**Authors:** Lorena I Orjuela, Martha L Ahumada, Ivonni Avila, Sócrates Herrera, John C Beier, Martha L Quiñones

**Affiliations:** Departamento de Salud Pública, Facultad de Medicina, Universidad Nacional de Colombia, Bogotá, DC Colombia; Grupo de Entomología Instituto Nacional de Salud, Bogotá, DC Colombia; Instituto Departamental de Salud de Nariño, Tumaco, Nariño Colombia; Caucaseco Scientific Research Center, Cali, Valle del Cauca Colombia; Department of Public Health Sciences, University of Miami Miller School of Medicine, Miami, FL USA

**Keywords:** *Anopheles calderoni*, Malaria, *Plasmodium falciparum*, *Plasmodium vivax*

## Abstract

**Background:**

*Anopheles calderoni* was first recognized in Colombia in 2010 as this species had been misidentified as *Anopheles punctimacula* due to morphological similarities. *An. calderoni* is considered a malaria vector in Peru and has been found naturally infected with *Plasmodium falciparum* in Colombia. However, its biting behaviour, population dynamics and epidemiological importance have not been well described for Colombia.

**Methods:**

To assess the contribution of *An. calderoni* to malaria transmission and its human biting behaviour and spatial/temporal distribution in the southwest of Colombia, human landing catches (HLC) and larval collections were carried out in a cross-sectional, entomological study in 22 localities between 2011 and 2012, and a longitudinal study was performed in the Boca de Prieta locality in Olaya Herrera municipality between July 2012 and June 2013. All mosquitoes determined as *An. calderoni* were tested by ELISA to establish infection with *Plasmodium* spp.

**Results:**

Larvae of *An. calderoni* were found in four localities in 12 out of 244 breeding sites inspected. *An. calderoni* adults were collected in 14 out of 22 localities during the cross-sectional study and represented 41.3% (459 of 1,111) of the collected adult specimens. Other species found were *Anopheles albimanus* (54.7%)*, Anopheles apicimacula* (2.1%)*, Anopheles neivai* (1.7%), and *Anopheles argyritarsis* (0.2%). In the localities that reported the highest malaria Annual Parasite Index (>10/1,000 inhabitants) during the year of sampling, *An. calderoni* was the predominant species (>90% of the specimens collected). In the longitudinal study, 1,528 *An. calderoni* were collected by HLC with highest biting rates in February, May and June 2013, periods of high precipitation. In general, the species showed a preference to bite outdoors (p < 0.001). In Boca de Prieta, two specimens of *An. calderoni* were ELISA positive for *Plasmodium* circumsporozoite protein: one for *P. falciparum* and one for *Plasmodium vivax VK*-210. This represents an overall sporozoite rate of 0.1% and an annual entomological inoculation rate of 2.84 infective bites/human/year.

**Conclusions:**

This study shows that *An. calderoni* is a primary malaria vector in the southwest of Colombia. Its observed preference for outdoor biting is a major challenge for malaria control.

## Background

*Anopheles calderoni* is a member of the Arribalzagia Series, which was described for the first time from material obtained from females captured in Salitral Department of Piura in Peru [1]. This species has also been reported in other areas in Peru [2, 3], in Venezuela [4] as well as Ecuador [5]. In Colombia, Gonzalez and Carrejo [6] included this species in the taxonomy key for the determination of *Anopheles* of Colombia with distribution data based on the review of several sources including samples from the Malaria Eradication Programme placed in the Entomological Museum collection of the Universidad del Valle (MUSENUV) and new specimens collected by Gonzalez and Carrejo during 10 years preceding the publication of the taxonomic key [6]. Later, González et al. [5] examined museum specimens from the Universidad del Valle using morphological character analysis of isofamilies. This combined with DNA sequence analysis of the second internal transcribed spacer (ITS2) and mtDNA cytochrome C oxidase subunit I gene (COI) barcodes (658 bp of the COI gene) showed that *An. calderoni* was present in 12 Colombian States. This also demonstrated that *An. calderoni* had been misidentified as *Anopheles punctimacula* due to similarities in the adult female characteristics of these two species and because the most common morphological keys for *Anopheles* determination used in Colombia [7, 8] did not include *An. calderoni* [9].

Females of *An. calderoni* can be taxonomically determined and differentiated from *An. punctimacula* by the presence of the following characteristics: upper mesanepimeron with pale scales, wings with pale yellow scales mixed with white scales, distal postsubcostal pale spot on vein C generally not contiguous with the postsubcostal pale on vein R1, separated (generally) or not by dark scales of the distal part of the postsubcostal dark and the proximal part of preapicaldark on R1; scales on vein R1 between the proximal part of the presubcostal pale and the distal postsubcostal pale generally dark, i.e., R1 in the subcostal area is usually all dark except for pale spots at the ends [1, 5].

Studies conducted in Colombia with *An. calderoni* are relatively recent. Gonzalez et al. indicated in 2010 the presence of *An. calderoni* in 22 municipalities of different states in Colombia (Antioquia, Bolivar, Caldas, Cauca, Chocó, Guajira, Magdalena, Nariño, Norte de Santander, Quindio, Tolima and Valle del Cauca). Additionally, studies conducted in three localities, Otoño, Candelaria and La Laguna de Sonso in Buga in Valle del Cauca demonstrated that this species displays a high heterogeneity in human biting activity that varies between unimodal and bimodal depending on location and abundance [9]. Finally, a recent study found *An. calderoni* infected with *Plasmodium falciparum* in Pindales municipality of San Andres in Tumaco in Nariño suggesting that this is a suspected vector [10].

In Peru, due to population abundance and the identification of *Plasmodium* infected mosquitoes, *An. calderoni* is considered a malaria vector in the western part of the country [1, 2].

Nariño State located in the southwest of Colombia is considered to be at high risk for malaria transmission with an average annual parasitic index (API) of ten and 9,010 malaria cases per year between 2004 and 2013 with 81.5% of cases due to *P. falciparum*, 18.3% due to *Plasmodium vivax,* and less than 1% due to *Plasmodium malariae* [11]. In previous records of *Anopheles* species in Nariño [6] 15 species were identified: *Anophelesalbimanus, Anophelesapicimacula, Anophelesargyritarsis, Anophelesboliviensis, An. calderoni, Anopheleseiseni, Anopheles forattini/costai, Anophelesmalefactor, Anophelesneivai, Anophelesneomaculipalpus, Anophelesoswaldoi, Anophelespseudopunctipennis Anopheles punctimacula, Anophelesrangeli* and *Anophelestriannulatus.* By observation or detection of *Plasmodium* parasites by salivary gland dissection, immunoradiometric and enzyme-linked immunosorbent assays or the coincidence between geographic distribution and malaria transmission, many of these species are considered malaria vectors in Colombia including: *An. albimanus,An. punctimacula, An. pseudopunctipennis, An.neivai, Anophelespholidotus (*as *Anopheleslepidotus)* [12]*, An.oswaldoi,* and *An.rangeli* [13–15]. However, given the morphological similarities between *An. calderoni* and *An. punctimacula*, the former has been confused with *An. punctimacula* in Colombia [5] and it is likely that the importance of *An. calderoni* as malaria vector in the southwest region of Colombia (Nariño State), as well as in other areas in Latin America, has not being recognized. The purpose of this study was to evaluate the epidemiological importance of *An. calderoni* in the southwest of Colombia where this species is present.

## Methods

### Study area

**Figure 1:**
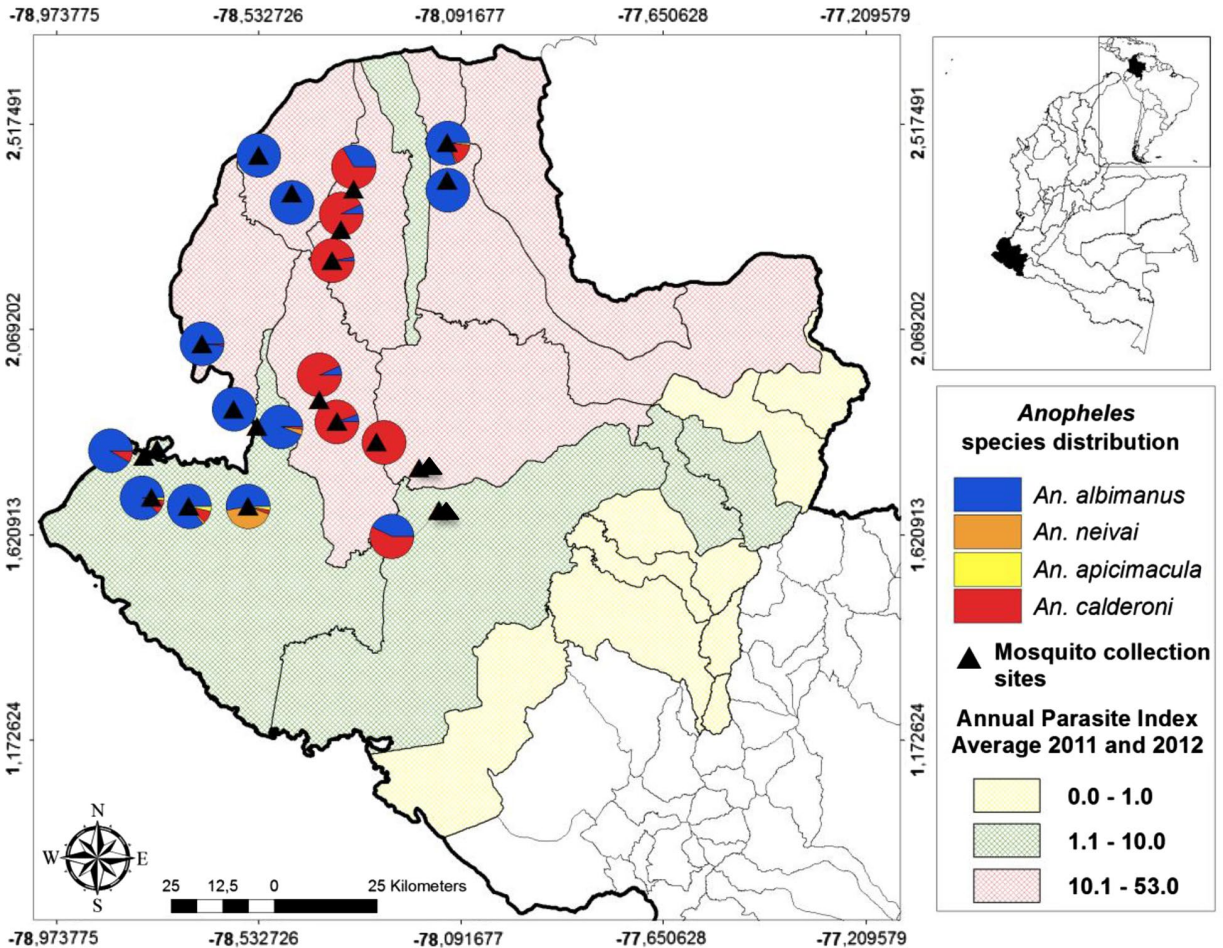
*Anopheles* species distribution in Nariño State, related to the Annual Parasite Index of each municipality. The precise locations names of the 22 sampled sites are in Table 1.

The southwest region of Colombia, Nariño State, is considered one of the highest malaria-endemic areas in Colombia. It is located on the border with the Republic of Ecuador and has an area of 33,268 sq km representing 2.9% of the national territory [16]. Administratively, it is divided in five sub-regions; the localities chosen for this study are situated in the Pacific Coast sub-region (Figure 1).

Briefly, the sub-region of the Pacific Coast is characterized as an area of difficult access and serious social and public disorder. The most important economic activities of this sub-region are based on agriculture, mainly African palm crops, fishing, mining, and emerging tourism. Ethnographically, the sub-region is composed of indigenous and Afro-Colombians [17]. The 22 selected localities (Table 1) are characterized by different levels of historical malaria incidence and due to their accessibility were selected for a cross-sectional study between May 2011 and August 2012. *Anopheles calderoni* was present with highest abundance in Boca de Prieta during the cross-sectional study and was selected to carry out a longitudinal study with monthly sampling between July 2012 and June 2013.

**Table 1:** Selected localities and number of adults and larvae collected in the cross-sectional and longitudinal studies in Nariño State

Municipality	Locality	Longitude	Latitude	m.a.s.l	*An. calderoni*	
					Adults	Larvae
Barbacoas	La Humildad	1.67371	−78.14091	52	4	
	Teraimbe	1.67414	−78.14044	48		
El Charco	Banguela	2.39497	−78.12092	22		
	Estero Martínez	2.47674	−78.12149	7	13	2
Magüí Payán	Brisas de Hamburgo	1.76708	−78.18384	48		
	Gualpi Piragua	1.76731	−78.18398	55		
Mosquera	Alto Guandipa	2.36366	−78.45803	11		
	Cocal Jiménez	2.44926	−78.53426	9		
Olaya Herrera	Boca de Guava	2.37614	−78.32665	9	2	
	Boca de Prieta (1)	2.28671	−78.35395	6	230	6
	Boca de Prieta (2)				1528	10
	San José la Turbia	2.21789	−78.37253	20	104	
Roberto Payán	Bocas de Telembí	1.82800	−78.27700	3	27	
	Chimbuza	1.91513	−78.40142	26	14	
	Pumbi Las Lajas	1.86900	−78.36300	3	17	
Salahonda	La Playa	2.03836	−78.65717	6	1	
Tumaco	Bucheli	1.70203	−78.76661	9	38	
	Ciudadela	1.79188	−78.78430	11	2	
	Colorado	1.85700	−78.53696	14	1	
	Curay	1.89530	−78.58761	13		1
	El Morrito	1.80894	−78.75467	3		
	Guayabo	1.68314	−78.55677	45	1	
	Robles	1.68457	−78.69072	3	5	39

*m.a.s.l* m above sea level, *1* cross-sectional study, *2* longitudinal study.

### Mosquito collections

#### Cross-sectional study

Breeding sites were inspected in each locality using a standard ladle with ten samples/sq m. Larval habitat type (excavations, fish pond, pool, lagoon, bromeliads, stream, or ditch) and temporary or permanent status were recorded. Adult mosquito collections were carried out during a week in each locality, as a unique observation. Adults were collected using human landing catches (HLC) indoors and outdoors simultaneously in eight houses for four consecutive nights from 18:00 to 24:00 hours by a two-person team with rotation every hour among collectors. All captures were made during the first 50 min of every hour. Mosquitoes were separated by location (indoors and outdoors), date, time and collector and kept dry over silica gel until processing.

#### Longitudinal study

Mosquito collections were carried out during a week each month, for 12 months, for a total of 48 collection nights in Boca de Prieta (municipality of Olaya Herrera, Nariño State). Mosquitoes were collected and stored as described above. In April and May 2013, the collections were carried out between 1800 and 0600 hours for eight nights. Those months were selected due to the empirical knowledge of the local technicians, in which during the first semester of each year both malaria cases and mosquito densities are higher. Particularly April and May were selected due to logistic reasons to have hired enough technicians for the 12-h collections. Houses in which HLC was carried out were selected, taking into consideration history of positive malaria cases in the last year, presence of mosquitoes during a previous survey and authorization of the residents and subsequent written informed consent by the head of household.

### Taxonomic determinations

Adult and larvae mosquitoes were determined using the most recent morphological key for *Anopheles* of Colombia [6]. Confirmation of the morphological determination of a sample of adults from each locality was done by COI sequencing. The DNA template was extracted from individual abdomens of mosquitoes using the DNeasy Blood & Tissue Kit (Qiagen). The amplified region of the COI mitochondrial gene was obtained using the primer pair of LCO 1490 (5′-GGTCAACAAATCATAAAGATATTGG-3) and HCO 2198 (5′-TAAACTTCAGGGTGACCAAAAAATCA-3) described by Folmer et al. [18] and following the protocol described by Ruiz et al. [19]. Sequencing reactions were performed using an Applied Biosystems 3500 Genetic Analyzer^®^ (PE Applied Biosystems), BigDye Terminator V 3,1^®^ kit and the Sanger dideoxy method (Sequencing and Analysis Service Molecular Genetics Institute—SSiGMol, Bogotá, Colombia). Sequences were aligned with the GenBank and Bold System databases using the Basic Local Alignment Search Tool [20, 21] to observe the pairing and identity with the most similar sequence.

### *Anopheles calderoni* distribution

The geographic location of each sampling site was determined using a global positioning system (GPS GARMIN 60CSX) and the points were visualized using the software ArcGis 9.0. The API in each municipality was illustrated as a layer in the map for the purpose of conducting analysis of species distribution in relation to the API of malaria in the state.

### Natural infectivity

Enzyme linked immunosorbent assay (ELISA) for *P. falciparum* and *P. vivax* (VK 210 and VK247 variant epitopes) circumsporozoite protein (CS) was conducted following standard protocols [22, 23] and using the ELISA kit distributed by the Centers for Disease Control (CDC, Atlanta, Georgia, USA). Only the head and the anterior part to the junction of the thorax and abdomen, between the middle and hind coxa of each mosquito [24], was tested by ELISA to avoid false positives inflating the entomological inoculation rate (EIR). Samples were tested in a 96-well ELISA plate along with seven negative controls which were laboratory-reared mosquitoes of *An. albimanus* Cartagena strain, and two positive controls corresponding to pure CS protein. The cut-off used was two times the average of the negative control [25]. Positive mosquitoes were retested using ELISA to increase specificity.

### Climatic data

Monthly temperature, precipitation and humidity data, recorded between 2002 and 2012, were provided by the Instituto de Hidrologia, Metereología y Estudios Ambientales de Colombia (IDEAM). Data from station No. 51035020 CCCP PACIFICO located in the Tumaco State were used for the analysis.

### Malaria cases

Data on the malaria cases of *P. falciparum* and *P. vivax* registered between 2003 and 2013 were gathered from the national surveillance system [Sistema Nacional de Vigilancia en Salud Pública (SIVIGILA)] [11]. The geometric mean of the malaria cases by month between 2003 and 2013 were represented graphically and correlated with the mosquito abundance and climatic data.

### Data analysis

The 12 h human-biting activity was calculated as the geometric mean of the mosquitoes found by hour, in the 8 nights of sampling in the longitudinal study. The sporozoite rate was estimated as the proportion of CS-positive mosquitoes divided by the total number of mosquitoes assayed. The annual EIR, defined as the number of bites by infectious mosquitoes per person per unit time, was expressed as the product of the number of bites/person/year and sporozoite rate [26] and was calculated only for the locality of Boca de Prieta in which the longitudinal study took place. Statistical analyses were performed using analysis of variance (ANOVA) and Student’s *t* test to test differences in mosquito counts between hours, months and sites (indoor and outdoor). *P* values ≤0.05 were considered statistically significant. Differences among collection months were compared using Duncan’s multiple-range test. To estimate the correlation between meteorological variables, mean monthly temperature, relative humidity, and precipitation and the abundance of mosquitoes (calculated as geometric mean), a multivariate linear model was carried out in which the data recorded for the same month or lagged by 1, 2 and 3 months. To estimate the relation between the number of cases and the abundance of mosquitoes, three linear model were estimated lagging mosquito abundance by 1, 2 and 3 months. All data were analysed using R statistical software (version 3.10).

### Ethical considerations

This study was approved by the National Institute of Health (NIH) DMID 11-0038 (longitudinal study) and 11-0046 (cross-sectional study) and the ethical committee of the Universidad Nacional de Colombia CE 079 and CE 078, respectively, Act No. 11 of June 20, 2011.

## Results

A total of 202 potential breeding sites were inspected of which 72.8% were excavations, 14.6% pools, 3.9% lagoons, 3.0% fish ponds, 3.0% ditches, 2.2% streams, and 0.4% other; of these, 83% were classified as permanent and 17% as temporal breeding sites. Larvae of *An. calderoni* were found in 12 breeding sites located in four localities: Estero Martinez, Boca de Prieta, Curay and Robles (Table 1). The breeding sites found with *An. calderoni* larvae were mainly human-made excavations (75%) used for domestic purposes (56%), all of which were permanent.

A total of 1,987 (1,528 longitudinal and 459 cross-sectional) adult *An.calderoni* mosquitoes were collected by HLC in 14 out of the 22 localities sampled (Table 1). At least one specimen of this species from each locality was confirmed by molecular methods. Seventeen COI sequences matched with four published sequences: two sequences matched (99.69% homology) the sequence HQ642974.1 from Guayas, Ecuador; two sequences matched (100%) the sequence HQ642971.1 from Guayas, Ecuador; eight sequences matched the sequence HQ642973.1 from Guayas, Ecuador: one with 99.39%, two with 99.69%, four with 99.85%, and one with 100% homology; and five sequences matched the sequence HQ642968.1 from Valle del Cauca, Colombia, three with 99.54% homology, one with 99.39% and one with 99.24%, confirming the identity of the collected samples as *An. calderoni*. Other species sampled, determined and confirmed were *An. albimanus* (54.7%), *An. apicimacula s.l.* [27] (2.1%), *An. neivai* (1.7%), and *An. argyritarsis* (0.2%).

### *Anopheles calderoni* distribution

*Anopheles calderoni* mosquitoes were found in 14 localities out of 22 sampled in the State of Nariño (Table 1). *An. calderoni* was found mainly inland between 25 and 30 km from the coast in two municipalities, Roberto Payan and Olaya Herrera, while *An. albimanus* predominated between 0 and 25 km from the coast (Figure 1). *An. calderoni* was predominant in the municipalities with API >10, the highest malaria transmission areas in the State, particularly in the municipalities of Olaya Herrera (average 2011–2012 API = 29) and Roberto Payan (average 2011–2012 API = 53) (Figure 1).

### Biting activity

**Figure 2:**
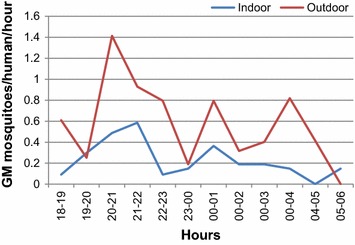
Biting activity of *Anopheles calderoni* in the locality of Boca de Prieta, Olaya Herrera, Nariño.

Twelve-hour sampling carried out in April and May 2013, for eight nights, was used for the analysis of biting activity of *An. calderoni* (n = 118). In general, the species showed human-biting activity all night, both indoors and outdoors (Figure 2). Despite increased activity observed between 20:00 and 21:00 hours in the outdoors and between 21:00 and 22:00 hours in the indoors, no statistically significant differences were observed in the collections made per hour by sampling sites (F = 0.7574; df = 11; p = 0.6807 indoor; F = 1.51; df = 11; p = 0.1414 outdoor). No significant difference (*t* = 1.32; p = 0.225) was found between the abundance during the first part of the night (18:00 to 0:00 hours) (58.5%) and the second part (0:00 to 6:00 hours) (41.5%). *An. calderoni* showed a statistically significant preference for outdoor (69.5%) *versus* indoor (30.5%) biting (*t* = −3.1251; *p* = 0.002).

### Monthly abundance

**Figure 3:**
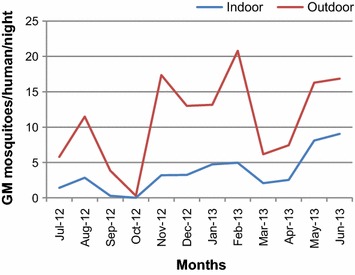
Monthly variation of *Anopheles calderoni* in Boca de Prieta, between July 2012 and June 2013.

The longitudinal study in Boca de Prieta showed variation in the abundance of mosquitoes collected monthly throughout the year. The maximum human-biting rate was observed in June 2013 with a peak of 12.4 bites per person per night (b/p/n) followed by February and May 2013 with 10.4 and 10 b/p/n, respectively (Figure 3). The highest indoor and outdoor abundance was observed in June and February as illustrated by Duncan analysis. A statistically significant preference in *An. calderoni* for outdoor (73.8%) *versus* indoor human-biting activity (F = 150.77; df = 1; *p* < 0.001) (Figure 3) throughout the sampling year was observed.

### Sporozoite rate and entomological inoculation rate

A total of 1,984 adult specimens were tested by ELISA resulting in two *Plasmodium* parasites positive *An. calderoni,* representing an overall sporozoite rate of 0.1%. The *Plasmodium* parasites species were *P. vivax* VK 210 and *P. falciparum* in mosquitoes collected indoors between 23:00 and 24:00 in September 2012 and outdoors between 22:00 and 23:00 in January 2013, respectively. The overall *An. calderoni* EIR was calculated as 2.84 infective bites/person/year. The EIR for each *Plasmodium* species would be 1.42 infective bites/person/year, as only one mosquito was positive for each *Plasmodium* species.

### Association between *Anopheles calderoni* abundance, climatic variables, and malaria morbidity

**Figure 4:**
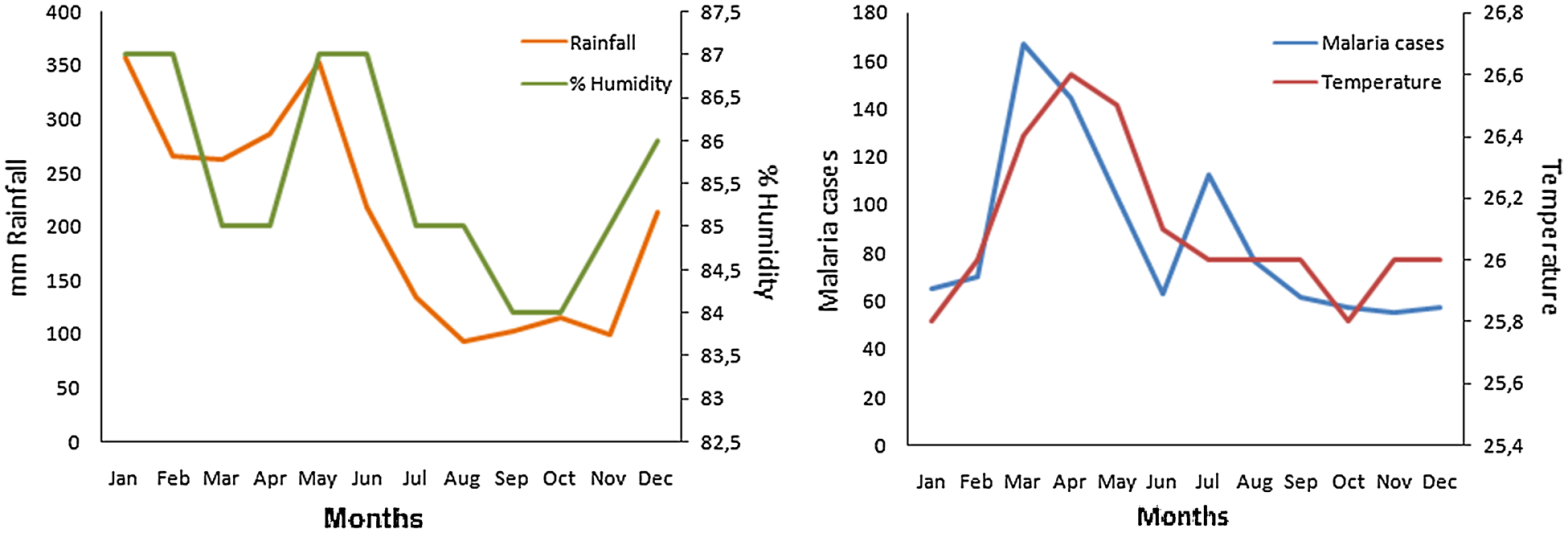
Monthly record of climatic variables reported by IDEAM between 2002 and 2012.

Figure 4 shows the climatic data (temperature, relative humidity and rainfall) recorded by Instituto de Hidrologia, Metereologia y Estudios Ambientales (IDEAM) at the station located in Tumaco municipality, and the geometric mean number of cases registered between 2003 and 2013 in Nariño State. The multivariate linear regressions showed that the environmental factors which best predicted the abundance of *An. calderoni* throughout the year were relative humidity and rainfall registered for the same month of sampling, which explained 90% of the variability in the model (R^2^ = 0.90, F = 48.5, *p* ≤ 0.001). No statistically significant association was found between the abundance of *An. calderoni* and malaria cases even considering climatic data lagged by 1 and 2 months. In the municipalities where *An. calderoni* was found most abundantly (Olaya Herrera and Roberto Payan), non-lagged monthly temperature was the environmental factor best predicting malaria cases throughout the year.

## Discussion

*Anopheles calderoni* has not been reported as a primary or even secondary vector in Colombia partly due to the misidentification as *An. punctimacula*. In this study, *An. calderoni* was the most abundant species sampled by HLC in high malaria transmission areas and was found positive for *P. falciparum* and *P. vivax* Vk-210 CS thus suggesting that *An. calderoni* is a primary malaria vector in the southwest of Colombia.

The correlation between *An. calderoni* abundance and rainfall and relative humidity could suggest that breeding sites of this species are temporary and that increases in precipitation could generate favourable breeding sites for the species and consequently affect abundance. This explanation is consistent with reports by Forattini [28], which describe breeding sites of *An. punctimacula* in shaded, cold and small farms, in soil depressions caused by car tyres and in footprints of animals where rainwater collected, some of which could be breeding sites of *An. calderoni* given the confusion that appeared between these species prior to the description of *An. calderoni*. However, the most abundant breeding sites found in this study were human-made, permanent excavations used to provide water for domestic purposes. Thus, the association between rainfall and adult *An. calderoni* abundance is unclear as the influence of rainfall on the breeding site formation is not evident. A follow-up of those permanent and other temporary breeding sites would clarify this apparent association. According to Urrego and Del Valle [29], based on weather station data, the study area climate is governed by that of the southern hemisphere where the first semester is wetter than the second with peaks in May and June and ecologically dry months with less than 60 mm of very scarce precipitation.

According to the results presented here, the highest abundance of *An. calderoni* in the region was recorded during the rainy season in the first 6 months of the year. These results agree with those previously reported by Naranjo-Diaz et al. [10] where peak abundance corresponded to the highest rainy period in the southwest of Colombia in San Andres in Tumaco, where *An. calderoni* presence was associated with small irrigation channels in oil palm plantations and wells. Despite the limitations of the data which were not collected monthly to permit estimations according to time-dependent environmental parameters, Lucumi et al. [9] suggest that *An. calderoni* population density variation is related to precipitation patterns.

The higher relative outdoor monthly abundance and nightly biting activity *of An. calderoni* suggest greater human exposure to infected mosquitoes outdoors compared to indoors. These results are similar to those reported by Cruz et al. [3] where *An. calderoni* in high abundance exhibited higher exophagy in Santa Rita Baja in northwest Chao in the State of La Libertad, Peru. However, these results differ from those reported by Naranjo-Diaz et al. [10] where a significant indoor instead of outdoor biting preference of *An. calderoni* was observed in San Andrés in Tumaco, Nariño State, Colombia.

Regarding *An. calderoni* human-biting nocturnal activity, the sampling intensity in this study was limited to eight nights and only covering 2 months, so caution is needed in generalizing these results. However, the biting pattern found was similar to previous reports in Perú (2,3) and in Colombia (9,10). The majority of specimens were collected before the household went to sleep during the first hours of the night compared to the second part of the night, although this difference was not statistically significant. This pattern was similar to that reported by Cruz et al. [3] in Peru even though the greatest activity was seen in later hours between 22:00 and 23:00 outdoors and 23:00 and 24:00 indoors and to that reported by Calderon et al. [2] where increased activity was observed between 19:00 and 22:00. Similarly, Naranjo et al. [10] observed the highest biting peak between 20:00 and 24:00 hours; however, other studies in Colombia illustrated that *An. calderoni* biting activity may vary between unimodal and bimodal depending on abundance. In high abundance areas, a peak may be observed before midnight (between 21:00 and 23:00 hours) and another after midnight (between 01:00 and 4:00). These differences are attributable to species phenotypic plasticity [9].

*Anopheles calderoni* is a recognized malaria vector in different parts of Peru, which shares a border with Colombia, and in Colombia was recently reported infected with *P. falciparum* [10]. *An. calderoni* infected with *P. falciparum* and *P. vivax VK* 210 during months when malaria incidence exceeded historical averages was observed in this study. The rate of infection reported here (0.1%) was similar to that reported for primary vectors in other typical endemic areas of Colombia such as *Anopheles nuneztovari* (0.101%) and *Anopheles darlingi* (0.087%) in Antioquia [30] and to that of local vector *Anopheles benarrochi* B (0.132%) in Putumayo [31]. However, this rate of infection is lower compared to those reported for *An. calderoni* in Peru (0.26–5.4%) where it is considered as a secondary malaria vector [2].

The data used here to estimate *An. calderoni* EIR meet the criteria suggested by Hay et al. [32] and Beier et al. [33] of at least one year of monthly sampling. The EIR estimated for *An. calderoni* (2.84 bites/person/year) was similar to that reported for primary vectors in Colombia including *An. darlingi* (2.9) in Meta [34], *An. nuneztovari* (3.5–3.6) in Antioquia and Cordoba, and *An. darlingi* (3.7) in Cordoba [30]. This demonstrates that despite existing heterogeneity in ecology and transmission and the relatively low densities in these areas, that the estimated *An. calderoni* EIR is similar to that reported for other recognized primary malaria vectors in Colombia.

Historically in Colombia, the reported malaria vectors included *An. punctimacula* as a secondary vector [13]. However, in this study, *An. punctimacula* was not found but *An. calderoni*, which suggests that *An. calderoni* is the species that is actually involved in malaria transmission in the south of Colombia.

The *An. calderoni* outdoor biting-behaviour preference exhibited in these areas indicates the necessity of introducing additional tools targeting outdoor transmission in the region to decrease human-vector contact. Possible control measures include modification or treatment of larval habitats which has proven successful using methods such as *Bacillus thuringiensis* israelensis and *Bacillus sphaericus* [35, 36] other larvicides such as Spinosad [37], which have been approved under WHOPES [38] for the control of mosquitoes, or the use of repellents [39] providing protection to people active in the outdoors during high vector activity. Local evaluation of possible control measures to complement residual indoor spraying or long-lasting insecticide nets is recommended.

## Conclusions

This study describes the distribution, biting patterns, seasonal abundance, and natural infectivity of *An. calderoni* in the southwest of Colombia, a region on the Pacific coast with high malaria transmission. These data show that *An. calderoni* is a malaria vector in this area. This new information should be used to formulate appropriate malaria control interventions in the area. Given the *An. calderoni* preference for outdoor-biting behaviour during hours of human activity, vector control measures complementary to any indoor-targeted control measure such as indoor residual spraying or long-lasting insecticide nets should be introduced to protect those exposed before bedtime. These results should also be taken into account in assessing effectiveness of current control measures promoted by the national malaria control programme. The behaviour exhibited by *An. calderoni* in this area will affect the efficacy of such personal protective measures and would thus render them insufficient for transmission control.

## Abbreviations

API: Annual Parasite Index
Bp: base pairs
CDC: centers for disease control
COI: cytochrome C oxidase subunit I gene
CS: circumsporozoite
DNA: deoxiribonucleic acid
EIR: entomological inoculation rate
ELISA: enzyme-linked immunosorbent assay
HLC: human landing catches
IDEAM: Instituto de Hidrologia, Metereologia y Estudios Ambientales
IGAC: Instituto Geográfico Agustin Codazzi
MUSENUV: Entomological museum collection of the Universidad del Valle
SIVIGILA: Sistema Nacional de Vigilancia en Salud Pública
